# The effect of natural products in animal models of temporomandibular disorders

**DOI:** 10.1590/1678-7757-2020-0272

**Published:** 2020-07-24

**Authors:** Janaíne Prata OLIVEIRA, Fernando Kenji NAMPO, Marilia Trindade Santana SOUZA, Luana Mendonça CERCATO, Enilton Aparecido CAMARGO

**Affiliations:** 1 Universidade Federal de Sergipe Programa de Pós-graduação em Ciências Fisiológicas São CristóvãoSE Brasil Universidade Federal de Sergipe, Programa de Pós-graduação em Ciências Fisiológicas, São Cristóvão, SE, Brasil.; 2 Universidade Federal de Integração Latino-Americana Instituto Latino-Americano de Ciências Naturais Foz do IguaçuPR Brasil Universidade Federal de Integração Latino-Americana, Instituto Latino-Americano de Ciências Naturais, Foz do Iguaçu, PR, Brasil.; 3 Universidade Federal de Sergipe Programa de Pós-graduação em Ciências da Saúde São CristóvãoSE Brasil Universidade Federal de Sergipe, Programa de Pós-graduação em Ciências da Saúde, São Cristóvão, SE, Brasil.

**Keywords:** Temporomandibular joint disorders, Biological product, Systematic review, Preclinical drug evaluation

## Abstract

Treatment of temporomandibular disorders (TMD) is a challenge for health care professionals. Therefore, new approaches have been investigated, such as the use of natural products.

**Objective:**

This systematic review aims to summarize the natural products used in treatment of experimental models of TMD.

**Methodology:**

A systematic search was performed in the databases Medline, Web of Science, Scopus, Embase, SciELO, LILACS, and Scholar Google databases in January 2020, dating from their inception. Pre-clinical studies with natural products for intervention in experimental TMD were included. Two reviewers independently selected the studies, extracted the data, and evaluated the risk of bias.

**Results:**

17 records were selected, and 17 different natural products were found, including three lectins, three plants or algae extracts, three sulfated polysaccharides, three cocoa preparations, and five isolated compounds. Concerning the risk of bias, most studies lacked on randomization and blinding. Nociception induced by phlogistic agents was evaluated in most articles, and in five studies it was associated with analysis of inflammatory parameters. In order to investigate the mechanism of action of the natural products used, eight studies evaluated expression of neural or glial molecular markers.

**Conclusions:**

16 of 17 natural products found in this review presented positive results, showing their potential for treatment of TMD. However, the lack of methodological clarity can influence these results.

## Introduction

Temporomandibular disorders (TMD) are multifactorial disorders that impair the temporomandibular joint, muscles of mastication, and muscle innervation of head and neck.^1,2^ These disorders approximately affect 5% of the population with a prevalence between 7.3% and 30.4% in the young people population and it can greatly affect the patients’ quality of life.^1,3^ The symptoms vary from mild discomfort to debilitating pain.^1,3^

The treatment of TMD is initially composed of the reduction of pain and debilitation, which can be achieved by several interventions with multidisciplinary approach. Conservative intervention includes patient education, physiotherapy, psychosocial management, occlusal splints and medicine. The association of these approaches is common in clinical practice.^4^ Generally, pharmacological approach, by itself or associated with other approaches still the main clinical approach.^5^ However, the effectiveness of the available medicines for TMD is questionable and their side effects can limit their clinical use.^5,6^ Therefore, other alternatives have been investigated, such as natural products (NP).

Natural products can include mixed substances present in plants and animals or even compounds isolated from them. Historically, they are an important source of active substances with therapeutic potential to treat several diseases, including inflammatory and painful conditions.^7^ Few clinical trials have evaluated natural products in patients with TMD. For example, a preliminary study showed that avocado/soybean extract decreased pain, improving the quality of life of patients with temporomandibular joint (TMJ) degeneration.^8^ The lack of clinical evidence on the use of NP to treat this condition reinforces the need for pre-clinical investigation.

Some pre-clinical studies evaluated the effects of NP in different experimental models of TMD.^9,10,11^ However, to the best of our knowledge, no study has systematically evaluated the use of natural products in experimental models of TMD. Thus, considering the need for new pharmacological alternatives to treat TMD, this study reviewed the use of different NP, essentially plant preparations or compounds isolated from plants, in pre-clinical studies involving animals submitted to models of TMD, to answer which NP (plant preparations and isolated compounds) have already been investigated for the treatment of experimental models of TMD.

## Methodology

This systematic review was carried out following the Cochrane Collaboration Handbook and PRISMA Statement recommendations.^12,13^

### Identification and selection of studies

#### Eligibility Criteria

Types of study: Pre-clinical studies comparing the efficacy of NP and innocuous intervention (e.g., saline or other vehicle) for the treatment of TMD.

Types of animals (participants/conditions): Animals submitted to any experimental model of TMD. Studies that evaluated animals with associated dental comorbidities were excluded.

Types of intervention: Studies comparing the beneficial effects of NP (plant preparations and isolated compounds) with any innocuous treatment. There was no limitation regarding dosage, timing, duration of the treatment, frequency or administration route. Studies involving combined treatments were excluded.

Types of outcome measures: Primary outcomes were orofacial nociception measured either by nociceptive behavior time or by the head withdrawal threshold, and/or expression of mediators, measured by immunohistochemistry assessment. A secondary outcome was inflammation, measured by myeloperoxidase activity or histology of structures involved in the pathology of TMD (temporomandibular joint, masticatory muscles, trigeminal nerve).

## Information Sources

Studies were identified by searching the following electronic databases: MEDLINE via PubMed, Web of Science, SCOPUS, EMBASE, Scientific Electronic Library Online (SciELO), Latin American and Caribbean Literature of Information in Health Sciences (LILACS), and Google Scholar. Handsearching with reference lists of included articles was also performed. No limits for language or country of publication were applied. All electronic databases were searched from inception.

## Search

**Figure 1:**
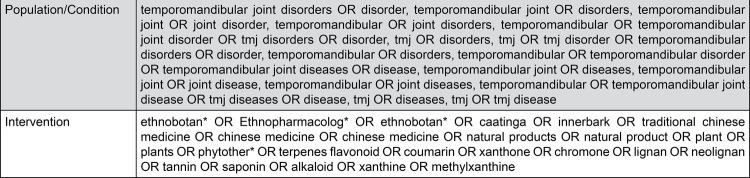
Search strategy

Studies were identified in January 2020. Pre-clinical studies that compared the efficacy of NPs with innocuous intervention were searched using the PICO framework. Based on the scarcity of titles found in our preliminary search, the search was not limited to pre-clinical studies, so synonyms of temporomandibular disorder were used as Population/Condition and synonyms of natural products were used as Intervention. The complete search strategy used for MEDLINE and adapted to the other databases is reported in Figure 1.

## Study Selection

After the identification of studies by the main researcher (JPO), two independent reviewers (JPO, MTSS) carried out the screening and eligibility analysis of the retrieved records. Discrepancies were resolved either by discussion or by a third reviewer (FKN).

## Data extraction

Two independent reviewers (JPO, MTSS) extracted data from the included studies in a unblinded manner using a structured table with information about authors, title, year of publication, language, animal characteristics (species, strain, age and weight), number of animals per group, TMD induction technique, intervention characteristics (type of NP, dosage of NP, duration of treatment, frequency and route of administration, and timing relative to TMD induction), and control characteristics.

Data extraction was performed following this sequence: (i) extract data from text or tables, (ii) extract data from figures (data labels) and then (iii) contact the correspondent author for unpublished data. Other authors were randomly contacted if no answer was obtained within a week or there was no contact information. If no answer was received after three weeks, the study was excluded from the analysis.

## Risk of Bias of Individual Studies

The evaluation of bias of the included studies was assessed by applying the SYRCLE’s Risk of Bias tool.^14^ Two independent reviewers (JPO, MTSS) assessed the internal validity of individual studies. The domain “another risk of bias,” was evaluated according to whether the study assessed or provided information about locomotor evaluation of the NP tested, the period in which the experiments were performed and if animals were previously habituated to the experimental setting.

## Results

### Search results

**Figure 2:**
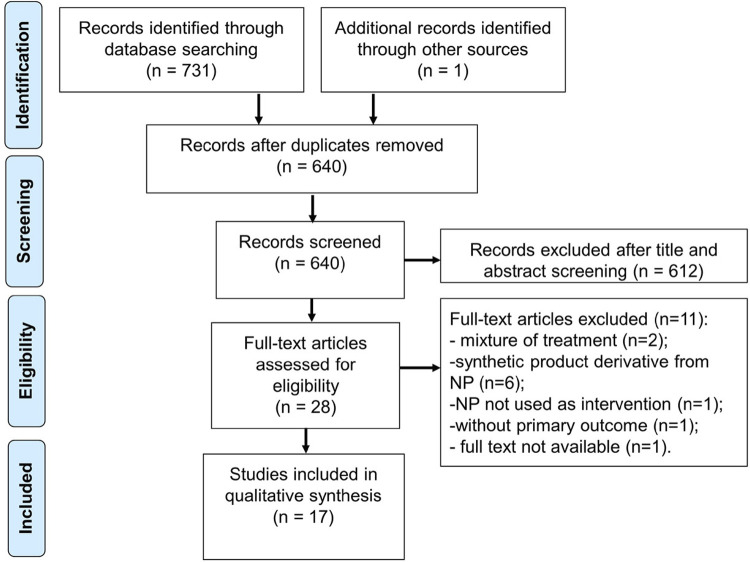
Flow chart of literature search

The electronic database search yielded 731 records: 246 from MEDLINE; 89 from SCOPUS; 253 from EMBASE; 11 from Web of Science; 9 from LILACS; 23 from SciELO, and 100 from Google Scholar. After excluding duplicates and including results of handsearching (n=1), 640 records remained. A total of 612 studies were excluded after title and abstract screening, then, 28 studies remained, of which 11 were excluded after full-text analysis for they did not present the review criteria. Thus, 17 studies were included in the qualitative synthesis (Figure 2).

### Characteristics of included studies

**Figure 3:**
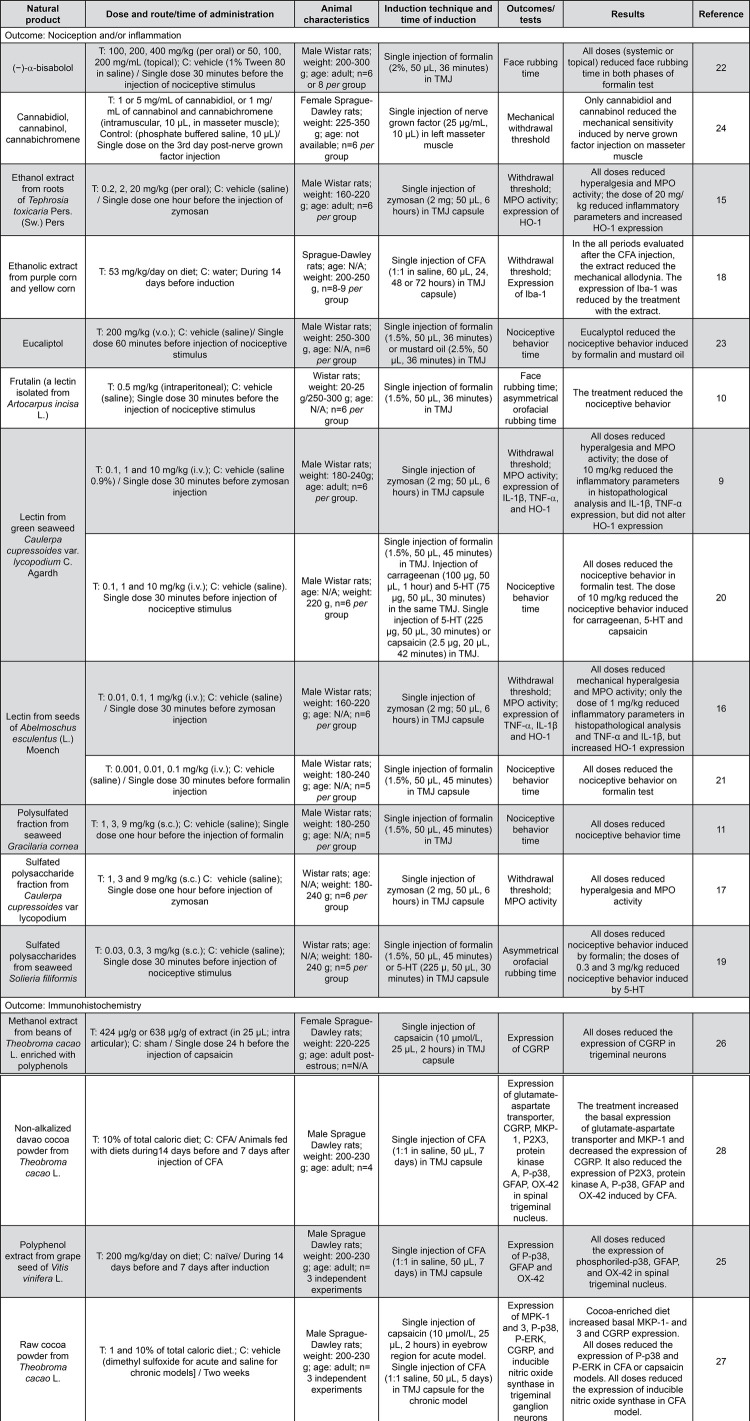
Characteristics of the studies included

Out of the 17 included studies, 14 described the use of plant and algae preparations, including crude extracts (4 studies), sulfated polysaccharide (3 studies), powder (2 studies), and lectins (5 studies); and three used isolated compounds. All studies were performed with rats and 76.47% of these evaluated nociception as an outcome, while inflammation was measured by 4 studies, and 5 studies assessed the expression of markers for neural or glial activation. Further details about the included studies are presented in Figure 3.

### Risk of bias assessment

**Figure 4:**
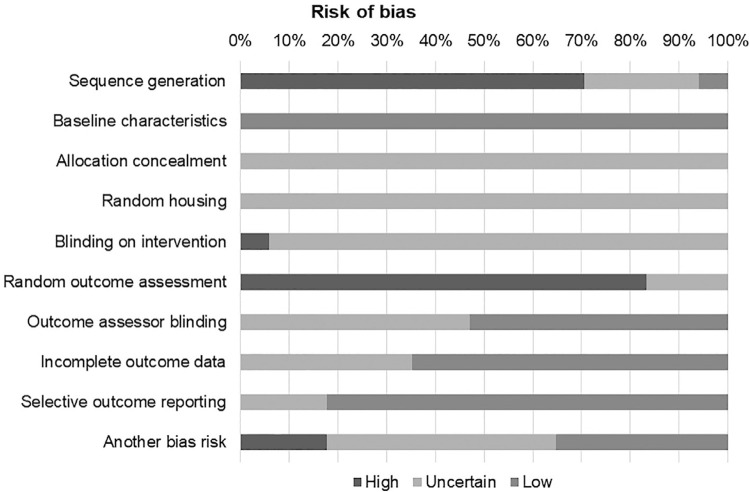
Risk of bias of the selected studies

Figure 4 shows that all included studies presented low risk of bias to ‘baseline characteristics,’ but no study presented low risk of bias to ‘allocation concealment,’ ‘random housing,’ ‘blinding for intervention,’ or ‘random outcome assessment.’ Only one study presented low risk of bias to ‘sequence generation.’ At least 50% of the studies presented low risk of bias for ‘outcome rater blinding’ (8 studies), ‘incomplete outcome data’ (11 studies) or ‘selective outcome reporting’ (14 studies).

### Effects of interventions – Outcomes

#### Nociception

In total, six studies evaluated nociception by measuring the effect of a mechanical stimulus in the orofacial region. They showed that intervention with ethanol extract from roots of *Tephrosia toxicaria*,^15^ lectins isolated from *Caulerpa cupressoides*^9^ or from *Abelmoschus esculentus* seeds,^16^ the sulfated polysaccharide fraction from *C. cupressoides*^17^, and the purple extract^18^ reduced nociceptive behavior induced by algesic stimuli in rats.

Seven studies investigated the effect of natural products on animals injected with formalin in the TMJ. The treatment with sulfated polysaccharides originated from the seaweed *Solieria filiformis*,^19^ with polysulfated fraction of the seaweed *Gracilaria cornea*,^11^ lectins isolated from *Artocarpus incisa*,^10^*C. cupressoides*^20^ and *A. esculentus*,^21^ (−)-α-bisabolol (topical or systemic)^22^ or eucalyptol reduced formalin-induced nociception in the TMJ.^23^ Four studies investigated NP in zymosan-induced nociception. These studies showed that ethanol extract from roots of *T. toxicaria*,^15^ lectins from the green seaweed *C. cupressoides*^9^ and seeds of *A. esculentus*^21^, and the sulfated polysaccharide fraction from *C. cupressoides*^17^ reduced nociception induced by zymosan. One study used nerve growth factor (NGF) to induce nociception in masseter muscle. In this study, cannabidiol and cannabinol, but not cannabichromene, reduced the mechanical sensitivity caused for this model.^24^

## Inflammation

Only five studies evaluated inflammatory parameters associated with the orofacial nociception. These studies used the zymosan model injection in TMJ to induce joint inflammation and associated nociception.

Thus, the treatment with the ethanol extract of roots of *T. toxicaria*,^15^ a lectin from *C. cupressoides*,^9^ a lectin from *A. esculentus*^16^ and the sulfated polysaccharide fraction of *C. cupressoides*^17^decreased myeloperoxidase activity and leukocyte counts in synovial lavage. This effect was accompanied by a reduction of histological alterations induced by zymosan in the TMJ for the highest dose of each study, with the exception of one study^17^ in which this analysis was not performed.

## Immunohistochemistry assessment

In order to characterize some mechanisms underlying the effect of the ethanol extract from *T. toxicaria,* the study by Val, et al.^15^ (2014) showed that the treatment with this extract increased the expression of heme oxygenase-1 (HO-1) in the model of zymosan-induced TMJ inflammation and nociception. In the same experimental model, the treatment with a lectin from *A. esculentus*^16^ reduced TNF-α and IL-1β expression, whereas it increased HO-1 expression. Interestingly, the treatment with the lectin from *C. cupressoides*^9^ reduced the expression of IL-1β and TNF-α, without altering the expression of HO-1.

In the model of CFA-induced TMJ inflammation, the polyphenol extract of grape seed (*Vitis vinifera* L.) reduced the expression of phosphorylated p38, glial fibrillary acidic protein (GFAP, a marker of activated astrocytes), and OX-42 (a marker of microglia activation) in trigeminal ganglia neurons when compared to animals injected with CFA.^25^ This model was also used by Magni, et al.^18^ (2018) that observed reduction in Iba-1 positive staining in trigeminal ganglia promoted by purple corn extract.

Three studies used *Theobrama cacao* L. as intervention against TMD in rats. Pre-treatment with methanol extract of *T. cacao* beans enriched with polyphenols decreased CGRP expression in trigeminal neurons after capsaicin injection in TMJ.^26^ In another study, the inclusion of cocoa powder in the diet in TMD induced by capsaicin or CFA models reduced the mitogen-activated protein kinase (MAPK) and inducible nitric oxide synthase (iNOS) expression and it also increased MAP kinase phosphatases (MKP) 1 and 3 expression in the trigeminal nerve.^27^ Besides, the inclusion of non-alkalized davao cocoa powder in the diet of CFA-injected rats bilaterally increased the expression of phosphorylated p38, GFAP, and OX-42 in the spinal trigeminal nucleus.^28^

## Discussion

Although the classification of Research Diagnostic Criteria for TMD is divided into four groups (temporomandibular joint disorders, masticatory muscle disorders, headache and associated structures),^29^ in this review, natural products were only tested in models of TMJ disorders and in masticatory muscle disorders. However, these two groups are the most prevalent types of TMD.^30^ In most cases of TMJ disorder, inflammation occurs in the synovial membrane of TMJ. The analysis of TMJ of patients with this disorder revealed a massive production of cytokines, chemokines and other inflammatory mediators. This inflammatory process is responsible for the pain and other signs and symptoms associated with disorders.^31,32^ In agreement with these findings, most studies identified in this review used phlogistic agents to induce inflammation in the synovial membrane, but different purposes can be identified since many studies only evaluated nociception, although others assessed the inflammatory process by using protocols involving zymosan or CFA lasting from induction for measuring neuronal or glial markers. Finally, NGF injection caused mechanical nociception in 3 days.

Different types of NP were found for the treatment of TMD, such as lectins, extracts of plants or algae, sulfated polysaccharides, powder preparations or isolated compounds that presented protective effects in models of experimental TMD.

Lectins bind to sugars present in the cell membrane, causing biological changes. This property may support the treatment of inflammatory and painful diseases.^33^ Three different lectins (frutalin, a lectin from *A. esculentus* seeds and another from green seaweed of *C. cupressoides*) were described regarding their ability to reduce nociception and inflammation in TMJ. The antinociceptive activity of frutalin depends of a nitrergic mechanism and it also modulates TRPA1 and TRPM8 channels.^10^ On the other hand, interestingly, the lectin from *A. esculentus* increased HO-1 and reduced TNF-α and IL-1β expression.^16^ In fact, HO-1 expression was associated with anti-inflammatory actions.^34^ Alves, et al.^21^ (2018) also showed that the treatment with this lectin reduces TNF-α concentration and it is opioid-dependent. For the treatment with *C. cupressoides* lectin no alteration of HO-1 expression was detected in TMJ, in spite of the results of a previous study showing that this lectin inhibited the immunoreactivity of HO-1, cytokines (IL-1β, IL-6 and TNF-α) and cyclooxygenase-2 in rat paws injected with carrageenan.^35^ A HO-1 expression-dependent mechanism was also observed based on the treatment with *T. toxicaria* extract after the injection of zymosan in TMJ.^15^

Polysaccharide sulfated from seaweed also presented beneficial effects in TMD models. The treatment with the sulfated polysaccharide fraction from *C. cupressoides* caused antinociception followed by reduced leukocyte infiltration in zymosan-injected TMJ,^17^ whereas the polysulfated fraction of *G. cornea* in the formalin model was described as dependent on opioid receptors and nitric oxide pathways as well as HO-1 activation and formation of IL-10 in the trigeminal ganglion and subnucleus caudalis,^11^ which corroborated previous results about this fraction down-regulated the production of proinflammatory mediators associated with the involvement of the HO-1 pathway^36^ The antinociceptive action of sulfated polysaccharides from *S. filiformis* also involved opioid receptors with increased release of β-endorphin in the subnucleus caudalis, while in TMJ the plasma protein extravasation and TNF-α and IL-1β concentrations were lower.^19^

Cocoa preparations were investigated in three studies. In the model of capsaicin-induced nociception, the treatment with methanol extract of cocoa beans reduced CGRP expression in the trigeminal ganglion.^26^ The CGRP is a sensorial vasoactive neuropeptide released in response to sensory fiber stimulation and it is involved in peripheral and central sensitization in TMD.^37^ The study by Cady and Durham^27^ (2010) also showed that the activation of p38 and ERK in the trigeminal ganglion neurons after CFA or capsaicin injection in TMJ was reduced by the treatment with cocoa powder incorporated in the diet of rats, and that iNOS expression was reduced in the CFA model. Finally, it was demonstrated that cocoa powder reduced the activation of PKA and p38 in neuron and glia along with the expression of GFAP and OX-42 in the spinal trigeminal nucleus,^28^ indicating that the cocoa treatment affects the activation of both neurons and glia. In fact, glial cells have relevant participation in nociceptive response modulation in trigeminal ganglia and nuclei and contribute to neuronal excitability and chronic inflammation.^32^ These results suggest that regular cocoa intake is beneficial in the treatment of TMD.

Furthermore, the treatment with ethanol extract from purple corn also affected microglia, as detected by reduced Iba-1 expression and shift of cell polarization to an anti-inflammatory phenotype after 1 to 3 days of CFA injection in TMJ.^18^ Attenuation of microglial activation in spinal trigeminal nuclei was also found for the treatment with polyphenol extract from grape seeds (*Vitis vinifera*) after 7 days of CFA injection in TMJ.^25^ Grape seed extract has been proposed as a promising anti-inflammatory agent, particularly due to its anthocyanidin content,^38,39^considered as a class of compounds that can reduce neuroinflammation.40

Interestingly, few isolated compounds were tested in TMD models since only (-)-α-bisabol, eucalyptol, and three cannabinoids were tested in models of formalin-induced nociception in TMJ. The mechanisms underlying these effects must be better elucidated. Eucalyptol is described as an agonist of TRPM8 leading to anti-inflammatory effects_,_^41^ a finding that corroborates its effects in a TMD models. On the other hand, (-)-α-bisabol effects were suggested to be partly dependent on TRPA1,^22^ although this remains unconfirmed. Cannabidiol and cannabinol are cannabis-derived compounds largely known to produce their effects by modulating the cannabinoid receptors pathway.^24,42^

### Strengths and limitations

The strength of this systematic review is related to the theme, since no review has evaluated the efficacy of NP in animal models of TMD. It also collected information in many databases, without restriction on language or publication. The lack of methodological rigor and detailed information is one limitation of the studies included. However, these methodological deficiencies are common in pre-clinical studies.^43^ Other relevant issue is related with the injection of phlogistic agents to TMJ partially mimics the alterations found in clinic conditions, but can provide important information for translational research. The lack of investigation about possible adverse events caused to NP in TMD models in most studies included is also a limitation. These issues can influence success in investigation of new treatment approaches.

### Future research and conclusions

A considerable number of natural products have already been tested against innocuous treatment in animal models of TMD. All of them presented positive results in the evaluated outcomes, mostly by reducing nociception, inflammation or glial activation. However, the lack of methodological clarity may impair the validity of the results. Therefore, future studies in this field should consider some details of experimental design, such as:

- Randomization of animals in the cages and in experimental procedures, as well as performance and analysis of experiments with blind researchers;
- Reporting more details about procedures performed to enable reproducibility of the research;
- Reporting possible adverse effects of natural products used for the treatment of TMD, to encourage the use of natural products in more studies.
